# Natural Deep Eutectic Solvent Extraction of Bioactive Pigments from *Spirulina platensis* and Electrospinning Ability Assessment

**DOI:** 10.3390/polym15061574

**Published:** 2023-03-22

**Authors:** Rodrigo Martins, Cláudia Mouro, Rita Pontes, João Nunes, Isabel Gouveia

**Affiliations:** 1 Association BLC3—Technology and Innovation Campus, Centre Bio R & D Unit, 3405-155 Oliveira do Hospital, Portugal; 2 FibEnTech Research Unit, Faculty of Engineering, University of Beira Interior, 6200-001 Covilhã, Portugal; 3 BLC3 Evolution Lda, 3405-155 Oliveira do Hospital, Portugal

**Keywords:** green solvents, green chemistry, nanofibers, microalgae

## Abstract

The first ever nanofibers produced by the electrospinning of polyvinyl alcohol (PVA) and *Spirulina platensis* extracts are presented in this article. *Spirulina platensis* extracts were obtained by ultrasound-assisted extraction (UAE) using two different solvents: a glucose/glycerol-based natural deep eutectic solvent (NADES) and water. Through spectrophotometry analysis, it was possible to determine the pigment yield of the extractions for both extracts: phycocyanin = 3.79 ± 0.05 mg/g of dry biomass (DB); chlorophylls = 0.24 ± 0.05 mg/g DB; carotenoids = 0.13 ± 0.03 mg/g DB for the NADES/Spirulina extracts, and phycocyanin = 0.001 ± 0.0005 mg/g DB; chlorophylls = 0.10 ± 0.05 mg/g DB; carotenoids = 0.20 ± 0.05 mg/g DB for water/Spirulina extracts. Emulsions were formed by mixing the microalgae extracts in PVA (9%, *w*/*v*) at different concentrations: 5, 20, 40, and 50% (*v*/*v*). Electrospinning was carried out at the following conditions: 13 cm of distance to collector; 80 kV of applied voltage; and 85 rpm of electrode rotation. After the nanofibers were collected, they were checked under a scanning electron microscope (SEM). ImageJ was also used to determine fiber diameter and frequency. SEM results showed the formation of nanofibers for 5 and 20% (*v*/*v*) of NADES/Spirulina extract content in the electrospinning emulsions, presenting diameters of 423.52 ± 142.61 nm and 680.54 ± 271.92 nm, respectively. FTIR confirmed the presence of the NADES extracts in the nanofibers produced. Overall, the nanofibers produced showed promising antioxidant activities, with the NADES/Spirulina- and PVA-based nanofibers displaying the highest antioxidant activity (47%). The highest antimicrobial activity (89.26%) was also obtained by the NADES/Spirulina and PVA nanofibers (20%, *v*/*v*). Principal Component Analysis (PCA) revealed positive correlations between both the antioxidant and antimicrobial activities of the electrospun nanofibers, and extract content in the emulsions. Moreover, PCA also indicated positive correlations between the viscosity and conductivity of the emulsions and the diameter of the nanofibers produced.

## 1. Introduction

Microalgae have been recognized as rich raw materials since they are composed of a large plethora of compounds—proteins, polysaccharides, long fatty acids, and pigments—that have been applied over the years in different industries including cosmetics, human food, and energy [1]. Bioactive pigments are interesting compounds present in several microalgae species that have drawn much attention from researchers, mainly due to their unique bioactive properties and for their color.

For instance, phycocyanin is a blue pigment that can be found in *Spirulina platensis*, which is among diverse pigments such as chlorophylls, xanthophylls, and carotenoids (beta-carotene, zeaxanthin, and myxoxanthophyll) that can be found in microalgae species [2]. Taking into account the ambitious sustainable goals set by the United Nations [3], which aim at reducing the dependency of the world’s economy on synthetic materials, natural bioactive pigments are becoming more relevant in society. In order to obtain bioactive pigments, different extraction methods are employed. 

The extraction method used to obtain bioactive pigments is one of the most important factors in the production of new products, since they have an impact (positive or negative) on the environment [1]. Conventional extraction techniques such as maceration, Soxhlet, solid–liquid extraction (SLE), and liquid–liquid extraction (LLE), are characterized by high volumes of toxic solvents and long extraction times. To overcome the limitations of these conventional extraction methods, non-conventional techniques such as microwave-assisted extraction (MAE) and ultrasound-assisted extraction (UAE) have been studied for the extraction of bioactive pigments from several biomass sources [1].

Additionally, aqueous-organic solvents such as hexane, benzene, methanol, chloroform, petroleum ether, and acetone, which are linked to several problems such as toxicity, volatility, and flammability, are frequently used in the extraction techniques used to obtain bioactive compounds and pigments from natural matrices [4]. Therefore, these solvents can be harmful to the environment, operator, and consumer health. Hence, researchers have found new innovative solvents such as ionic liquids (ILs), deep eutectic solvents (DES), and natural deep eutectic solvents (NADES) to help improve the overall process’s sustainability [4].

ILs are salts that contain an organic cation and an organic or inorganic anion and exhibit favorable thermodynamic characteristics for extraction processes, such as low vapor pressure, thermal stability, and the capacity to extract a variety of organic and inorganic compounds. Although they present such qualities, they are not regulated by the Food and Drug Administration (FDA) and their effects on human health are not totally elucidated [4].

DES are formed by a hydrogen bond acceptor (HBA) and a hydrogen bond donator (HBD). DES present desirable thermodynamic properties for extraction processes, but due to some reported toxicity issues [5], they could also present some risk to human health.

Thus, NADES—DES made out of natural components—seem to bring the most safety to extraction processes while having the same thermodynamic advantages of a DES. Other major improvements such as: the ability to form transparent liquids at ambient temperature, which make these solvents easy to prepare and use; being non-toxic solvents; and being biodegradable make NADES an attractive solvent option. Since NADES are seen as less toxic and more biodegradable than the other two new generation solvents discussed (ILs and DES), they are a great and suitable solvent choice for the extraction of bioactive compounds and pigments from biomass sources (i.e., microalgae) [1].

In a recent study by Wils et al. [6], the first screening of NADES for the extraction of bioactive pigments and free fatty acids from *S. platensis* was reported. In their study, Wils et al. [6] used ultrasound to extract bioactive pigments (i.e., chlorophylls, carotenoids, and phycocyanin) from the microalgae. After initial screening of several NADEs, a glucose/glycerol-based NADES showed the highest phycocyanin yield. This was an important study, since it shed a light on NADES use in extraction processes using microalgae biomass. 

After extraction, the functionalization of the bioactive pigments is quite important. In this scope, techniques such as electrospinning play a major role in the production of nanomaterials with the incorporation of microalgae extracts.

Electrospinning (electrostatic fiber spinning) is a simple and effective method widely used in the field of nanotechnology, which uses electric fields to produce micro- and nanofibers [7,8,9]. The popularity of electrospinning rose during the end of the 20th century, when many publications started to appear and continue today, where many applications for electrospun fibers, such as wound healing, tissue engineering, and textiles, were discovered [9]. In addition, the electrospun fibers are very advantageous due to their flexibility and large surface area, making them suitable for medical and textile applications because of their superior waterproofing properties and breathability compared with thicker fibers [7].

Some studies have reported the use of the electrospinning technique with the incorporation of different types of biomass sources. For instance, Kim et al. [10] reported a nanofibrous dressing composed of a PCL/Alginate/Spirulina nanofiber by coaxial electrospinning. In the study, the authors extracted *Spirulina platensis* from Jeju lava sea water and stirred the microalgae species in distilled water for one day, prior to emulsion formation. Although this approach is quite interesting, the study failed to quantify the bioactive compounds and pigments present in the *S. platensis* extracts. In addition, the use of extraction techniques such as UAE could help enhance the extractability of bioactive compounds, without affecting the cell wall’s integrity [11].

Due to the antimicrobial properties of the microalgae-derived bioactive pigments [12], it is very important to analyze their potential use in several applications, more specifically in the formation of antimicrobial nanofiber-based materials, for applications in textiles, food-packaging, etc.

Antimicrobial textiles are functionally active textiles that may kill the microorganisms or inhibit their growth [1]. These antimicrobial textiles can be used in a wide variety of applications including air filters, health care, hygiene, medical, sportswear, storage, ventilation, and water purification systems [13]. Likewise, nanofiber-based materials for food packaging with antimicrobial function are at the basis of food preservation, which is also aligned with UN sustainability goals. 

In this study, a green and sustainable technology was assessed: freeze-dried *Spirulina platensis* was submitted to UAE, where a glucose/glycerol-based NADES was used. Then, the extracts were mixed with polyvinyl alcohol (PVA) to form an electrospinning emulsion. Therewith, the prepared emulsions were electrospun and the produced nanofibers checked under SEM and further characterized in terms of bioactive function. To the best of our knowledge, this is the first study where extracts resulting from *Spirulina platensis* NADES and UAE have been combined with PVA to form nanofibers through the electrospinning method.

## 2. Materials and Methods

### 2.1. Chemicals

D-glucose anhydrous (MW = 180.16 g mol^−1^) and glycerol (MW = 92.09 g mol^−1^) were purchased from Fisher Scientific (Hampton, NH, USA). ABTS (MW = 548.69 g mol^−1^) was purchased from PanReac (Barcelona, Spain). Polyvinyl alcohol (PVA) (MW = 115,000 g mol^−1^, Viscosity = 46–54 mPa.s at 20 °C) was purchased from VWR Chemicals (Leuven, Belgium). Potassium persulfate (MW = 270.3 g mol^−1^) was purchased from Acros Organics (Geel, Belgium).

### 2.2. Spirulina platensis Production

*Spirulina platensis* was maintained in Zarrouk culture medium in a thermostatic greenhouse at 25 ± 2 °C. The agitation of the culture and the supply of CO_2_ was carried out through the continuous injection of air. The light intensity was maintained at 12 μmol s^−1^m^−2^ and the photoperiod 12 h light and 12 h dark. *S. platensis* biomass was then obtained through vacuum filtration. Afterwards, the biomass was freeze-dried and kept in polypropylene flasks until further use.

### 2.3. Spirulina platensis Extraction

Freeze-dried biomasses were extracted using ultrasound and a glucose/glycerol/water-based (1:2:4 molar ratio) NADES during 30 min with biomass/solvent ratio (1/20, *w*/*w*). The resulting extract was then centrifuged for 10 min at 16,200 g (Rotanta 460R, Hettich). The supernatant was recovered and solid residue was extracted two more times (final biomass/solvent ratio 1/60). Each extraction was performed in triplicate. 

The same procedure was then used for water extraction of *S. platensis,* and served as a point of reference for all the analyses conducted throughout this research article.

### 2.4. Spectrophotometry Analysis of the Spirulina platensis Extracts

Spectrophotometry analysis was conducted in UV5, Mettler Toledo. For the analysis, the aliquots were filled with the extracts, then the highest absorbance at a specific wavelength spectrum was determined, and the concentration of bioactive pigments such as chlorophylls a and b, carotenoids, and phycocyanin were determined using Equations (1)–(3), respectively [14,15]:

(1) $$C_{chlorophyll\;a+b}\;({\mu g\;\mathrm{mL}}^{-1})=17.76A_{646.6}-7.34A_{663.6}$$

(2) $$C_{carotenoids}\;({\mu g\;\mathrm{mL}}^{-1})=4.69A_{440}-0.267\;C_{chlorophyll\;a+b}$$

(3) $$C_{phycocyanin}\;({\mathrm{mg}\;\mathrm{mL}}^{-1})=(A_{620}-0.474A_{652})/5.34$$

where $C_{chlorophyll\;a+b}$ is the concentration of chlorophylls a and b in μg/mL, $C_{carotenoids}$ is the concentration of total carotenoids in μg/mL, and $C_{phycocyanin}\;$ is the concentration of phycocyanin in mg/mL. In addition, $A_{646.6}$, $A_{663.6}$_,_ $A_{440}$, $A_{620}$, and $A_{652}$ are the absorbance values at 646.6, 663.6, 440, 620, and 652 nm, respectively.

The yield of each pigment was determined following Equation (4), also used by other authors [16]:

(4) $$Yield\;\left(\frac{\mathrm{mg}}{g}\right)=C_{pigment}\times \frac{V}{DB}$$

where $C_{pigment}$ is the concentration of chlorophylls a and b, carotenoids, and phycocyanin expressed in mg/mL; V is the volume of solvent expressed in mL; and DB is the dried biomass used in the extraction, expressed in g. 

### 2.5. Determination of Minimum Inhibitory Concentration (MIC)

Minimal Inhibitory Concentration (MIC) of the Spirulina extracts was determined against two bacterial strains: *Staphylococcus aureus* (ATTC 6538) and *Klebsiella pneumoniae* (ATCC 4352) by the broth microdilution method according to CLS M07-A6 guidelines. Briefly, the extracts were prepared with DMSO (10% (*v*/*v*)) to yield a concentration of 20 μL/mL. Then, sequential dilutions were prepared using MHB with concentrations of 1–10 μL/mL. Next, the overnight bacterial cultures were adjusted to 0.5 McFarland turbidity standards with sterile water. Afterwards, bacterial work suspensions were prepared using 500 μL of the 0.5 McFarland suspensions and 4500 μL of MHB. A volume of 50 μL of bacterial work suspensions and 50 μL of the diluted solutions of the extracts were added into 96 multi-well polystyrene plates (Sigma-Aldrich). The multi-well plates were incubated for 24 h at 37 °C, after which the 0.02% (*w*/*v*) resazurin solution was added to each multi-well plate to aid visualization of bacterial growth and further incubated for 4 h. Deposited bacteria in the bottom were evaluated. The color change of resazurin from blue to purple or pink was taken as the MIC value. Wells filled with MHB medium and bacterial suspension were used as a positive control (K+), whereas wells containing only MHB medium were used as a negative control (K−). All determinations were performed in triplicate.

### 2.6. Preparation of the Emulsions Used for Electrospinning

The electrospinning emulsions were prepared using the supernatant of the *Spirulina platensis* NADES and UAE and the co-spinning agent PVA (9%, *w*/*v*), with concentrations of 50%, 40%, and 20% (*v*/*v*). The prepared emulsions were then stirred in a magnetic shaker and heated at 110 °C until a homogenous emulsion was obtained. The emulsions were used in further procedures when the temperature of the emulsion was at room temperature.

### 2.7. Conductivity Measurement of the Emulsions

The conductivity of the emulsions prepared for electrospinning was determined using the HI2003-02, HANNA instruments (Woonsocket, RI, USA). For the assay, the electrode of the HI2003-02 device was submersed in the emulsions. The result was read on the display after stabilization. All determinations were performed in triplicate.

### 2.8. Viscosity Measurement of the Emulsions

A rotational viscometer (VR 3000 MYR, model V1-L, Viscotech Hispania SL.) was used to measure the viscosity of emulsions. The room temperature and the solution temperature were also measured before and after each viscosity measurement. For each solution, three different spindles (TL5, TL6, and TL7) were analyzed at different rotation speeds in order to accurately measure the viscosity of the emulsion. The viscosities of the emulsions were then read on the display of the VR3000 device. All determinations were performed in triplicate. 

### 2.9. Electrospinning

**Figure 1:**
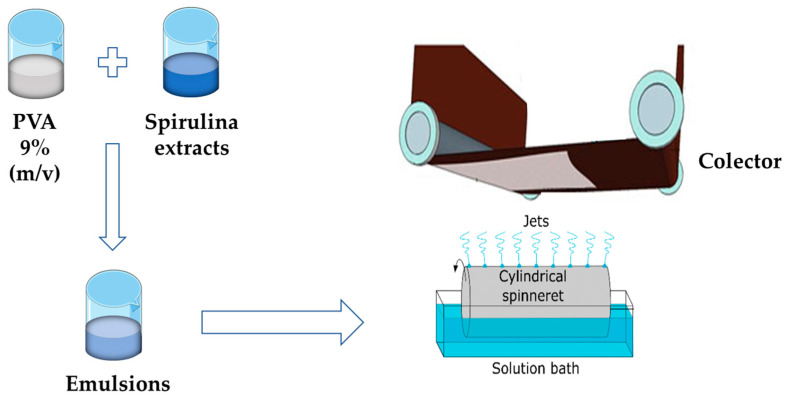
Electrospinning scheme.

The stable and homogenous emulsions were electrospun using Nanospider Technology (Nanospider laboratory machine NS LAB 500S from Elmarco s.r.o., Czech Republic, elmarco.com, accessed on 18 February 2023). Electrospinning of the prepared emulsions was carried out using the following conditions: 13 cm, 80 kV, 85 rpm. The collection time was set at 10 min. Experiments were conducted at room temperature (≈20 °C). The electrospun fibers were collected on polypropylene nonwoven fabric and dried at room temperature until constant weight. Figure 1 illustrates the electrospinning of the emulsions prepared with PVA and Spirulina extracts. 

### 2.10. Surface Electron Microscopy (SEM) of Electrospun Nanofibers

The electrospun fiber samples were mounted on aluminum stubs and then coated with gold using a Sputter Coater (Emitech). Then, the samples were examined using a Hitachi (S 2700) SEM at an accelerating voltage of 20 kV. ImageJ software (Image J, National Institutes of Health, Bethesda, MD, USA) was used to calculate average fiber diameter and diameter distribution from the SEM micrographs by randomly selecting 50 nanofibers. The results were expressed as the average diameter of the 50 nanofibers and were graphically illustrated as histograms.

### 2.11. Fourier Transform Infrared Spectroscopy (FTIR) of the Nanofibers

Fourier transform infrared spectroscopy (FTIR) was conducted in the IRAffinity-1S from Shimadzu, Japan. For the FTIR analysis, small sample sizes were cut randomly from the collector (polypropylene nonwoven fabric) used in the electrospinning technique, which contained the produced nanofibers. All the samples were then analyzed and cross-checked against the LabSolutionsIR (Version 2.11, Shimadzu, Kyoto, Japan) library. The scanning range was 4000–600 cm^−1^ with a resolution of 4 cm^−1^. Polypropylene nonwoven fabric was used as the background. 

### 2.12. Color Analysis of the Nanofibers

The color analysis of the nanofibers was conducted using a Datacolor 110 spectrophotometer (Datacolor company, Lawrenceville, NJ, USA) under illuminant D65 using 10° standard observer.

The colorimetric properties of the functionalized samples were evaluated in terms of CIELab and CIELch values (L*, a*, b*, C*, and h). L* indicates lightness from black to white (0 to 100, respectively); positive and negative values for parameter a* represent redness and greenness, respectively; C* indicates the saturation or purity of the color; and h represents 0° for redness, 90° for yellowness, 180° for greenness, and 270° for blueness, using polar coordinates. Color strength (K/S) was also determined according to Equation (5):

(5) $$\frac{K}{S}=\frac{{\left(1-R\right)}^{2}}{2R}$$

where R is the observed reflectance of the colored sample at 540 nm, K is the absorption coefficient, and S is the scattering coefficient.

### 2.13. Antioxidant Acitivity Determination by ABTS Assay

Antioxidant activity was evaluated by the ABTS radical decolorization assay as previously described by other authors [17]. Briefly, the ABTS radical cation (ABTS+) was produced by reacting 5 mL of ABTS (7 mM) stock solution with 88 μL of potassium persulfate (2.4 mM). The mixture was then stored at room temperature, in the dark, for 12–16 h before use. Prior to the beginning of the assay, the ABTS+ solution was diluted with phosphate buffer (0.1 M, pH 7.4) to reach an absorbance of 0.700 ± 0.025, at 734 nm. Then, 10 mg of each sample was added to 10 mL of ABTS+ solution, and the reaction occurred for 30 min in the dark. The scavenging capability of ABTS+ at 734 nm was calculated using Equation (6):

(6) $$Antioxidant\;activity\;\left(\%\right)=\frac{A_{control}-A_{sample}}{A_{control}}\times 100$$

where A_control_ is ABTS+ initial absorbance and A_sample_ is the absorbance of the remaining ABTS+ in the presence of the nanofibrous sample. All determinations were performed in triplicate.

### 2.14. Antimicrobial Activity of the Nanofibers

The ability of the nanofibers containing bioactive pigments extracted from *S. platensis* to inhibit *S. aureus* (ATCC 6538) and *K. pneumoniae* (ATCC 4352) growth was tested through Japanese Industrial Standard JIS L 1902:2002, a standard method widely used to test the antibacterial activity of textiles. Bacterial suspensions (1 ± 0.3 × 10^5^ CFU/mL) were prepared and inoculated over the nanofibers and a PVA (9%, *w*/*v*) sample, which acted as a control. The antibacterial activity was assessed immediately after inoculum application (T0h) and after 24 h (T24h) in contact with the agar slurries, at 37 °C. For this purpose, the samples containing the inoculum were subjected to vigorous vortex for 30 s in a neutralizing solution and serial dilutions were prepared with 0.85 (*w*/*v*) NaCl at T0h and T24h, spread on nutrient agar plated in agar plates, and incubated at 37 °C for 18–24 h at 37 °C, in order to determine CFU/mL. Then, the percentage of bacterial reduction (R) was calculated accordingly with Equation (7):

(7) $$R\;\left(\%\right)=\frac{\left(C-S\right)}{C}\times 100\;$$

where S represents the number of CFUs obtained with the nanofiber samples and C is the CFUs of bacteria recovered from alkaline-treated PVA (9%) control.

### 2.15. Nomenclature

In Table 1, the nomenclature used throughout the article for the extracts and the nanofibers obtained is summarized.

### 2.16. Statistical Analysis

The results were analyzed by one-way ANOVA analysis of variance technique, Pearson correlation test, and Principal Component Analysis (PCA) using Excels’ XLSTAT. The *p* values of less than 0.05 were considered statistically significant. Data were expressed as mean ± standard deviation value.

## 3. Results and Discussion

### 3.1. Pigment Determination of the Spirulina platensis Extracts

Due to the great properties (antimicrobial and antioxidant) of the bioactive pigments present in *Spirulina* extracts, the quantitative determination of such compounds is highly relevant. Thus, using spectrophotometry (UV-VIS), it was possible to determine the pigment concentration of the W/Sp and NADES/Sp extracts. Spectrums for both extracts are available for consultation in Figures S1 and S2 of the Supplementary Materials.

**Figure 2:**
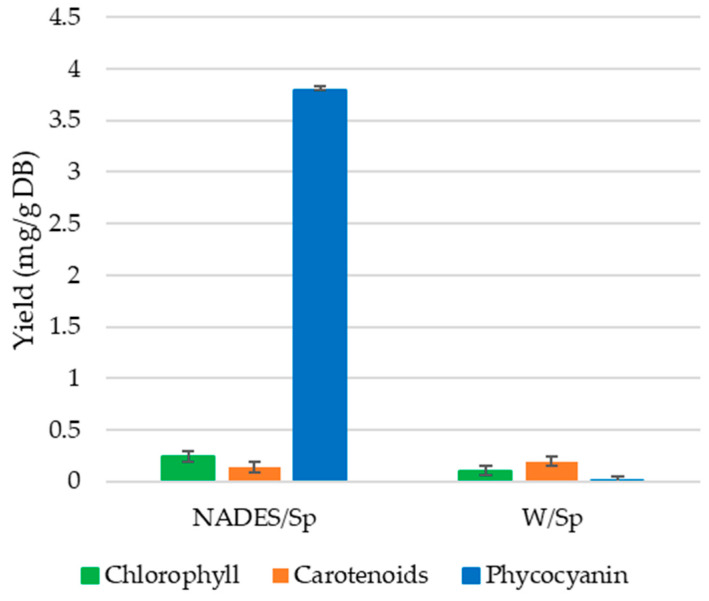
Yield (mg/g Dry Biomass) of W/Sp and NADES/Sp extracts.

In Figure 2, the yields of bioactive pigments of both W/Sp and NADES/Sp extracts are presented. From the results obtained, it can be drawn that phycocyanin yield (3.79 mg/g DB) was similar to the reported yield in the study by Wils et al. [6] (3.96 mg/g DB), while total chlorophyll content (0.24 mg/g DB) was lower than the results obtained in the study (0.69 mg/g DB) [6]. Total carotenoid content was also lower (0.13 mg/g DB) than what was reported by Wils et al. (0.22 mg/g DB).

On the other hand, phycocyanin yield (0.001 mg/g DB) was the lowest of the bioactive pigments obtained with ultrasound and water extraction, while the total chlorophylls (0.10 mg/g DB) were lower than that of the NADES/Sp extracts, and carotenoid content (0.20 mg/g DB) was higher. Thus, the ultrasound extraction using a glucose/glycerol-based NADES proved to be more efficient than ultrasound and water extraction in the extraction of phycocyanin and chlorophylls, while water and UAE was more effective in the extraction of carotenoids. 

Despite the positive results, extraction optimization might be missing in order to enhance the overall yield of the bioactive pigments, and consequently improve the antimicrobial and antioxidant properties of the NADES extracts.

### 3.2. Determination of the Minimum Inhibitory Concentration (MIC) of the Extracts

Minimum inhibitory concentrations (MICs) are defined as the lowest concentration of an antimicrobial agent that will inhibit the visible growth of a microorganism after overnight incubation [18].

*S. platensis*-derived pigments such as carotenoids, chlorophylls, and phycocyanin have already been studied for their antibacterial properties [19,20]. For instance, purified phycocyanin extracted from *S. platensis* showed a MIC of 50–500 μg/mL against *S. aureus* (Gram-positive bacteria) and other bacteria [21]. On the other hand, fucoxanthin (carotenoid derived from *S. platensis*) showed similar results against *S. aureus*, around 125 μg/mL, and 250 μg/mL against *K. pneumoniae* (Gram-negative bacteria) [19].

In Table 2, the MIC of both water and NADES extracts obtained from UAE of freeze-dried *S. platensis*, are shown. The results of the assay showed no standard deviation for both extracts. From the results obtained, it can be concluded that W/Sp extract showed a MIC of 10 mg/mL against both bacteria analyzed (*K. pneumoniae* and *S. aureus*), while NADES/Sp extract presented lower MIC values, 4.17 mg/mL against *K. pneumoniae* and 8.34 mg/mL against *S. aureus*. In addition, the results are in line with previous reports [22], where several solvents were used in the extraction of *S. platensis*, and the MIC was determined. In the study conducted by Usharani et al. [22], hexane extracts showed a MIC of 10 mg/mL against *K. pneumoniae* and *S. aureus* bacteria, while other organic solvents (i.e., ethanol, acetone, and methanol) showed lower MICs (≈5 mg/mL).

As previously mentioned, there have been significant research studies highlighting that less toxic and harsh solvents should be employed in extraction processes, following the main principles of green chemistry [4,23]. Hence, given the results obtained for the NADES/Sp extracts, NADES present a viable solvent alternative for the extraction of bioactive pigments from *Spirulina platensis*, since the extracts show similar MIC values as the organic solvent extracts reported in reference [22], while still being less toxic, and biodegradable [4,6,24].

### 3.3. Conductivity of the Electrospinning Emulsions

In the electrospinning technique, the repulsion of the charges at the surface of the solution causes the solution to stretch and form the nanofibers. The stretching of the electrospun jet and its bending stability are controlled by the Coulomb force between charges and by the force of the external electric field [25]. Both of these forces emerge due to the surface charge on the jet; hence, it can vary by changing the conductivity of the solution used in the electrospinning. In general, electrospun nanofibers with the smallest fiber diameter can be obtained from the solution with the highest conductivity [25]. 

**Figure 3:**
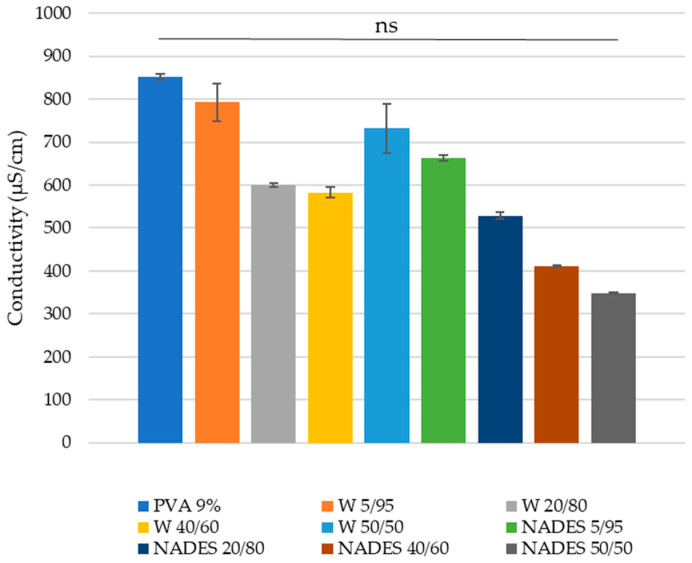
Conductivity (μS/cm) of the emulsions used to form nanofibers. ns—non-significant for *p*-value of 0.05.

The conductivity results of the emulsions used in the electrospinning technique are shown in Figure 3. As expected, PVA showed the highest conductivity, which is related to its ability to form nanofibers. In addition, the W 5/95 emulsions showed high conductivity, as expected due to the high content in PVA. Furthermore, for the NADES/PVA-based emulsions, conductivity decreased with lower PVA content. Although the same was not verified for the W/PVA-based emulsions, due to the higher conductivity of the W 50/50 emulsion, the W 40/60 showed lower conductivity than the W 20/80. 

The effect of the conductivity of the samples in the diameter of the nanofibers will be further discussed in this article.

### 3.4. Viscosity of the Electrospinning Emulsions

Solution viscosity is one of the main factors affecting the electrospinning technique, with too low viscous solutions usually leading to intermittent beads in the nanofiber structure, whereas a too high viscosity reduces the system processability [26]. Therefore, viscosity determination of the emulsions of *S. platensis* extracts and the co-spinning agent PVA is quite important.

**Figure 4:**
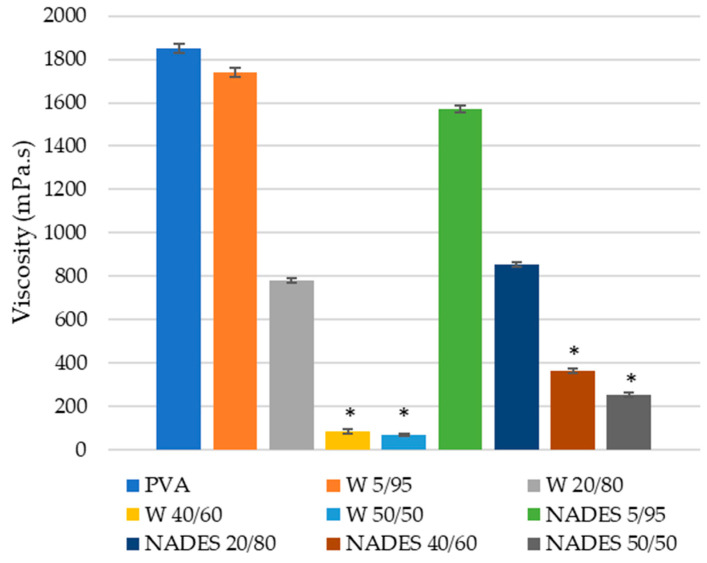
Viscosity (mPa.s) of the emulsions used in the electrospinning. *—statistical differences for *p* < 0.05.

The results obtained for the viscosity of the emulsions used in the electrospinning technique are illustrated in Figure 4.

As expected, the viscosity of the emulsions decreased with lower PVA content in the emulsions. Moreover, while the W 5/95 emulsion showed higher viscosity than the NADE 5/95 emulsion, with higher extract content in the emulsion, overall it became evident that the NADE/PVA-based emulsions showed higher viscosity than the W/PVA-based emulsions with the same relative extract to PVA content.

As studied by Briscoe et al. [27], PVA viscosity might be affected by the degree of hydrolysis, temperature, and pressure. For instance, at high degrees of hydrolysis, where inter- and intra-chain hydrogen bonding is dominant in the solutions, the PVA showed an increase in viscosity with storage time, due to the formation of strong chain entanglements and associations. For this case, apparent viscosity decreased with the increase in temperature and of pressure. On the other hand, at lower degrees of hydrolysis, where hydrogen bonding between the PVA chains and water molecules was more significant, the viscosity of the PVA solutions decreased with temperature due to the disruption of the hydrogen bonding between solute and solvent [27]. This is important, since it can affect the overall viscosity of the emulsions used in the electrospinning.

In this study, a high degree of hydrolysis PVA was used. At higher degrees of hydrolysis, the dissolution of PVA in water is more difficult, requiring higher temperatures, but on the other hand the polymer exhibits higher mechanical resistance when compared with PVA obtained with a lower degree of hydrolysis [28]. The effects of the emulsions’ viscosity in the diameter of the nanofibers produced through the electrospinning technique will be further discussed in this article.

### 3.5. SEM of Electrospun Nanofibers

In recent years, electrospinning has been widely used in the preparation of tissue engineered scaffolds because of its simplicity, economy, and high controllability of fiber diameters [29]. During the electrospinning process, several jets are continuously elongated towards the collector, the solvent of the polymeric jets is evaporated quickly, phase separation occurs, the jet solidifies, and nanofibers are formed [9]. More importantly, the electrospun nanofibers exhibit decreased fiber diameter when compared with the microfibers fabricated from some conventional spinning methods, such as melt spinning, wet spinning, dry spinning, and dry-wet spinning, thus resulting in notably increased specific surface area, which is highly needed in several medicinal applications [29,30]. 

Despite the great advantages of the electrospinning technique, there are several parameters that may impact fiber formation and morphology [31]. Nanofiber formation can be affected by: (1) the properties of the solution—viscosity, polymer concentration, electrical conductivity, and surface tension; (2) the processing conditions—applied voltage, distance from electrode to collector, and electrode type; and (3) the ambient conditions—temperature and humidity [31]. Therefore, electrospinning was conducted under several controlled conditions such as: electrode type, applied voltage, distance to collector, temperature, and humidity. 

Since microalgal extracts cannot be electrospun alone due to their weak rheological properties, spinnability, low mechanical strength, low solution viscosity, low surface tension, and low electrical conductivity, which cause the formation of unstable jets, combining synthetic or natural polymers as co-spinning agents is a great way to improve the electrospinning results [32].

In this study, PVA was the polymer used to form emulsions with W/Sp and NADES/Sp extracts. PVA is a synthetic biodegradable polymer that possesses great mechanical properties such as semi crystallinity and high temperature stability, which are very advantageous to the electrospinning technique [33,34]. Additionally, when compared with other polymer options often used in the electrospinning, PVA is relatively low cost [34]. Although PVA lacks some bio-functional capabilities, its mechanical and biodegradable features make up for such disadvantages. Additionally, such bio-functional capabilities may be mitigated with the incorporation of *Spirulina* extracts.

**Figure 5:**
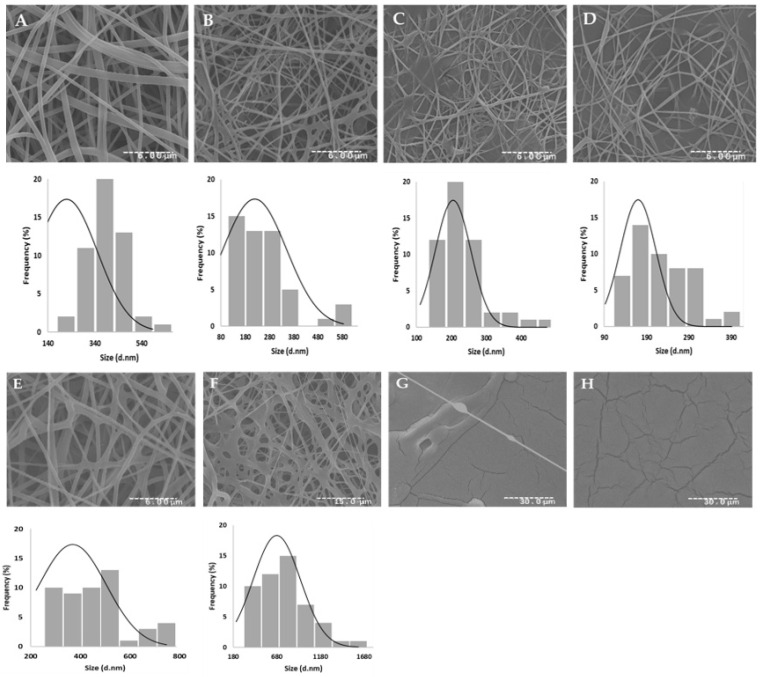
SEM analysis of the nanofibers produced: (**A**)—W 5/95; (**B**)—W 20/80; (**C**)—W 40/60; (**D**)—50/50; (**E**)—NADE 5/95; (**F**)—NADES 20/80; (**G**)—NADES 40/60; (**H**)—NADES 50/50, and the corresponding histograms of the diameter distribution obtained from ImageJ software.

SEM was used to analyze the morphology and formation of the nanofibers. The micrographs were then examined using ImageJ software in order to accurately measure the diameter size and fiber frequency. The results obtained in SEM and the diameter distribution fitted to the normal distribution curve are illustrated in Figure 5.

From the results, it was possible to conclude that W 20/80 nanofibers had a small fiber diameter and a smooth surface morphology. Additionally, with increasing extract content in the electrospinning emulsions (i.e., W 40/60 and W 50/50), nanofibers presented lower smoothness, but still presented thin fibers. When compared with the W 5/95 nanofibers, the W 50/50, W 40/60, and W 20/80 showed higher frequencies of smaller size fibers, although their morphology was less smooth and uneven.

On the other hand, NADES 20/80 fibers showed higher fiber diameter, but still kept a smooth morphology. The increased diameter of the NADES and PVA fibers might be linked to the ability of the NADES to form hydrogen links [35]. As a point of reference, NADES 5/95 nanofibers were checked under SEM, and due to the higher PVA content in the emulsion, the NADES 5/95 nanofibers presented high frequencies of thinner fibers. Nonetheless, with increased NADES/Sp extract content (i.e., NADES 40/60 and NADES 50/50 nanofibers), the results proved to be unpromising, given the non-formation of nanofibers. Thus, raising the content of NADES/Sp or W/Sp extracts in the electrospinning emulsions does not seem viable or favorable to fiber formation and morphology.

Although SEM results can be seen as unpromising, especially for the NADES/Sp-based nanofibers, it is important to mention that other reports [32,36,37,38] focused on the formation of nanofibers using *S. platensis* extracts, employed higher co-polymer concentrations in order to obtain smoother fibers. Moreover, different organic solvents (e.g., chloroform and methanol) are largely seen in use in the extraction processes prior to the emulsion formation, making the overall process less sustainable, more hazardous, and not suitable for in vivo applications [4,32].

Through this first screening, it was possible to evaluate the viability of the W/Sp and NADES/Sp extracts and PVA to form nanofibers via electrospinning. PVA-based nanofibers could be used in a wide range of biomedical applications, such as wound dressings, tissue scaffolds, and drug release, because of their solubility in water, biocompatibility, and biodegradability under specific conditions of PVA [39]. Thus, this could open new pads for greener extraction and production strategies that are in alignment with UN sustainability goals. Overall, SEM results were promising, and should be optimized in further research.

### 3.6. FTIR Analysis of the Nanofibers

FTIR analysis is a technique often used for the identification of organic, inorganic, and polymeric materials utilizing infrared light for scanning the samples. FTIR detects alterations in the characteristic pattern of absorption bands that clearly indicate a change in the material composition [40]. 

**Figure 6:**
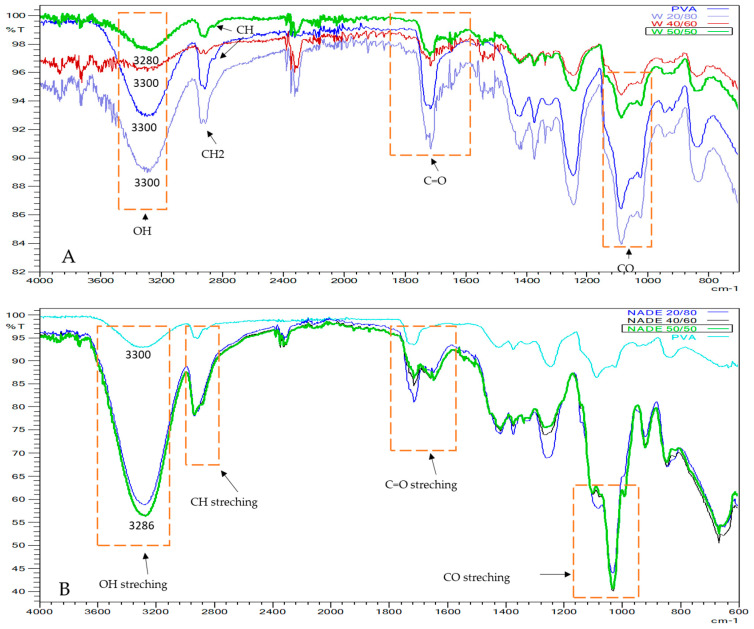
Spectrum for each nanofiber produced: (**A**) PVA, W 20/80, W40/60, and W50/50; (**B**) PVA, NADES 20/80, NADES 40/60, and NADES 50/50.

In our study, FTIR was used for the identification and characterization of compounds present in the nanofibers produced through the electrospinning technique. The FTIR spectra obtained are illustrated in Figure 6. 

The PVA sample showed main absorption peaks at: 3300 cm^−1^, attributed to OH stretching; 2862 cm^−1^, due to CH stretching; C=O stretching was found at 1739.61 cm^−1^; and 1093 cm^−1^ due to CO stretching [41]. Furthermore, the W 20/80, W 40/60, and W 50/50 also showed similar peaks as PVA.

The NADES/Spirulina/PVA-based nanofibers showed similar peaks as PVA and glycerol. The FTIR spectrum of the nanofibers was complex (Figure 6B), showing peaks of several functional groups: O-H stretching frequency was observed at 3286 cm^−1^; C=O stretching was found at 1739.61 cm^−1^, probably due to the esters present in the glycerol [42]; and C-O stretching of the primary alcohol was shown at 1112 cm^−1^. 

**Figure 7:**
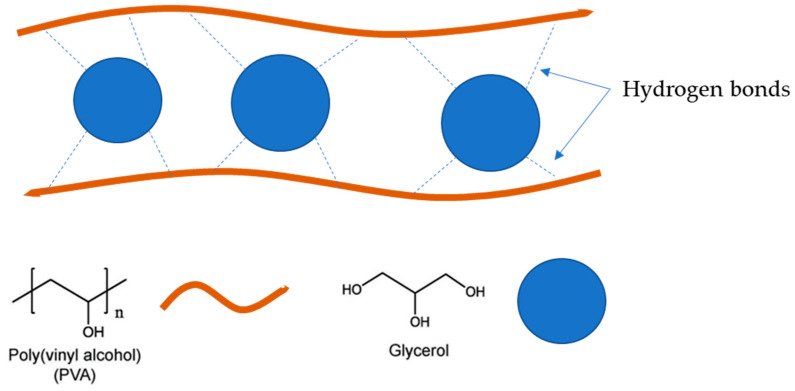
Schematic illustration of the H-bonding between glycerol and PVA.

The absorption peaks of OH stretching in the NADES/PVA-based nanofibers decreased with increasing NADES/Spirulina extract content. An abrupt and large shift from 3300 to 3286 cm^−1^ was observed when the extract content was increased. The shift of the absorption bands to lower wavenumbers suggests the formation of H-bonding between glycerol molecules and PVA chains, as proposed in Figure 7 [43]. Thus, with increasing NADES/Sp extract content in the emulsions used in the electrospinning, the FTIR spectrum showed similarities to glycerol. The same was also observed in the W 50/50 nanofibers, since OH stretching decreased from 3300 to 3280 cm^−1^; this might also be linked to the ability of the water molecules to form hydrogen bonds with PVA [33].

Therefore, FTIR analysis has shown differences between the NADES-based nanofibers and water-based nanofibers, which might be linked to the interaction between PVA and both solvents used in this study, meaning that extracts are likely present in the fibers, and that the solvents used helped the crosslinking of the nanofibers [44]. 

After cross-checking the spectrum data obtained for each sample with the LabSolutionsIR library (Table 3), PVA was found to be the most common match, while the NADE 40/60 and the NADE 50/50 nanofibers were matched with glycerol, probably due to the higher glycerol content present in these nanofibers. 

### 3.7. Color Analysis of the Nanofibers

Color analysis was conducted for all nanofibers produced. Table 4 summarizes the different CIELab and CIELch values obtained, as well as the apparent color of the nanofibers and color strength. However, the data showed no statistically significant differences between the nanofiber samples and the control sample (PVA 9%). NADES 40/60 and W 50/50 nanofibers displayed a slight green/blue color, allusive to the chlorophylls/phycocyanin extracted from *S. platensis*. While the extraction of pigments has not been optimized, the results show promise, since higher pigment concentration will probably enhance the color of the nanofibers, which might be an interesting feature in the development of nanofiber materials for applications in textiles, food-packaging, etc.

### 3.8. Antioxidant Activity Determination by ABTS Assay

ABTS+ radical scavenging assay is an electron transfer-based assay widely used for the assessment of the antioxidant capabilities of natural products. This spectrophotometric technique is based on the ability of compounds to donate hydrogen to free radicals; thus, the antioxidants reduce the ABTS+ radical to a colorless compound [17].

**Figure 8:**
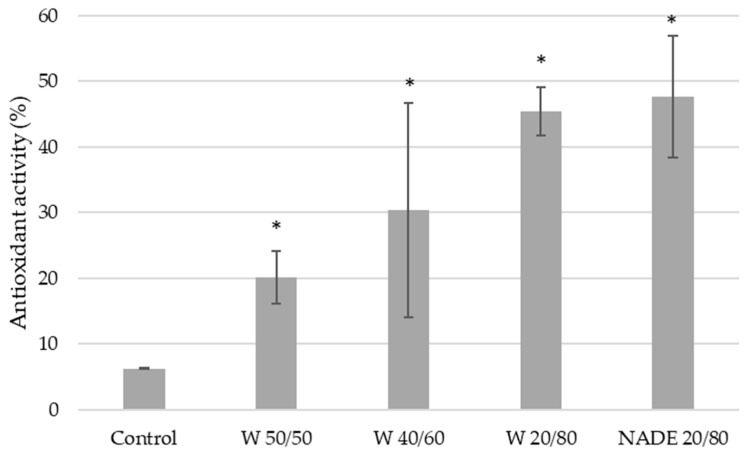
Antioxidant activity (%) of the electrospun nanofibers. *—statistical differences for *p* < 0.05.

The antioxidant activity of the nanofibers was analyzed, and the results are illustrated in Figure 8. The results showed significant differences (*p* < 0.05) compared with the control sample (PVA 9%), displaying higher antioxidant activity than the control sample. In addition, the NADES 20/80 nanofibers had the highest antioxidant activity (47%). The W/Sp- and PVA-based nanofibers showed higher antioxidant activity rising with higher PVA content in the emulsion.

Nonetheless, the results are promising, since other studies [45], focused on the fiber production with microalgae compounds through the electrospinning method, reported antioxidant activities of 19–32% while employing the ABTS assay, also used in this article. Moreover, Moreira et al. [45] highlighted the potential of the bioactive fibers for food preservation applications, and it was further mentioned by Karaduman et al. [32] in an important review focused on algal nanofibers.

Other studies [17] performed in our research group focused on the electrospinning of bacterial pigments for packaging applications, which also showed lower antioxidant activities (38.96 ± 0.49%) than those of the NADES/PVA-based nanofibers. Thus, the results obtained for the nanofibers functionalized with bioactive pigments extracted from *Spirulina platensis* in a green process using NADES and UAE show promising antioxidant capabilities that are valuable for packaging, textile, and pharmaceutical applications.

### 3.9. Antimicrobial Activity of the Nanofibers

The antimicrobial inhibitory activity of the functionalized nanofibers with active bioactive pigments from *S. platensis* was evaluated against two major bacteria often found in food—*S. aureus*—and in hospitals—*K. pneumoniae*. These bacteria were analyzed due to the assays’ requirement of a Gram-positive (i.e., *S. aureus*) and Gram-negative bacteria (i.e., *K. pneumoniae*); nevertheless, due to the plethora of applications that electrospun fibers have (packaging, wound healing, textiles, etc.), it is important to test the fibers against different types of bacteria that may occur in different environments and conditions. By doing so, it will give a better perspective of the antimicrobial capacity of the functionalized materials in different types of application fields.

**Figure 9:**
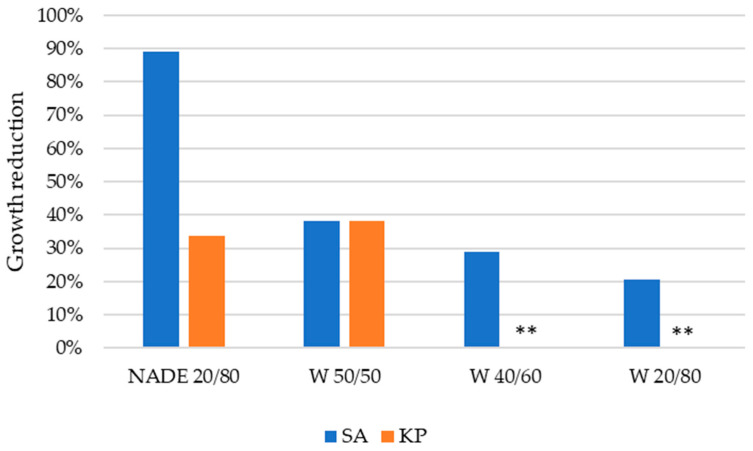
Growth reduction (%) of the *S. aureus* (SA) and *K. pneumoniae* (KP) bacteria obtained for the nanofibers produced. **—unable to measure.

The results obtained for the antimicrobial activity of the electrospun fibers are shown in Figure 9. The results indicate that the NADES 20/80 fibers proved to be most effective against *S. aureus*, showing an 89.26% reduction, while W 50/50 showed a significantly lower growth reduction of 38.23%. In addition, the growth reduction against the *S. aureus* bacteria was higher than that against the *K. pneumoniae* for all the nanofibers tested. Moreover, growth reduction decreased with higher extract content.

The higher antimicrobial activity of the NADES/Sp nanofiber is probably linked to the higher yield of bioactive pigments reached by the NADES and UAE extraction of *S. platensis*.

Despite the results obtained, it is important to point out that the indications of the US FDA and their European counterparts consider that antibacterial properties exist in the case of bacterial reduction of at least ≥99.99% [46]. Thus, the overall antibacterial potential of the nanofibers is considered subpar by the US FDA standards, even at 89.26% reduction—the highest reduction obtained for the NADES-based nanofibers. Therefore, more research must be put towards optimizing the antibacterial potential of PVA/Spirulina nanofibers.

### 3.10. Principal Component Analysis (PCA) and Pearson Correlation Test

**Figure 10:**
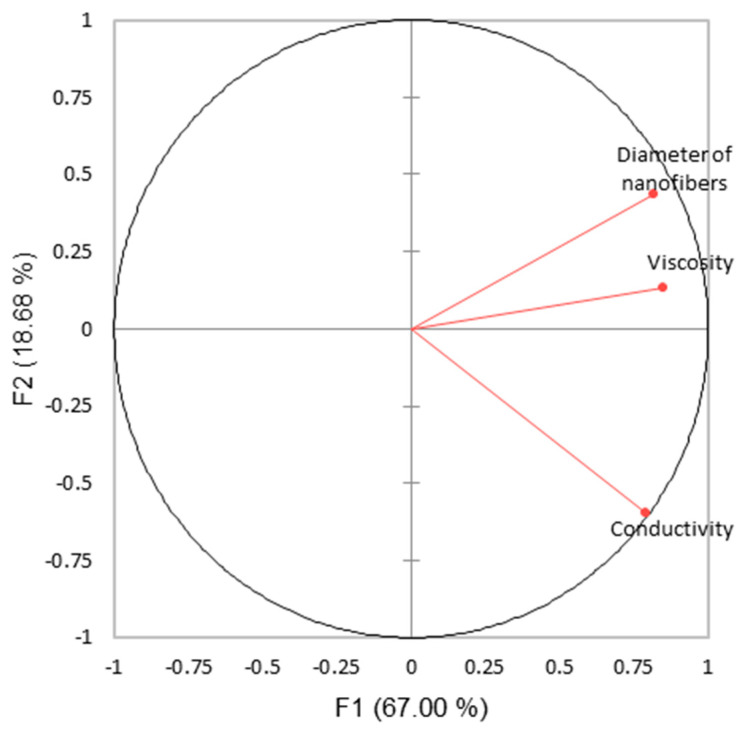
PCA biplot for the conductivity and viscosity of the emulsions and the diameter of the nanofibers.

Pearson correlation test and PCA were performed in order to evaluate significant relations between all of the emulsion variables tested, and the results obtained (i.e., diameter of the nanofibers, antioxidant activity, and antimicrobial activity). Therefore, the correlation between the emulsion properties (i.e., viscosity and conductivity) and the diameter of the nanofibers were firstly analyzed, since several reports already showed that these properties affect the diameter of the nanofibers [25,26]. The correlation matrix and PCA biplot are shown in Table 5 and Figure 10, respectively. 

From the PCA and Pearson correlation test, it can be drawn that both the viscosity and conductivity are positively related with diameter of nanofibers. Although the data are not statistically significantly correlated, the viscosity of the emulsions seems to be more influent than the conductivity of the emulsions in the diameter of the nanofibers. Thus, with high viscous emulsions, higher diameter of nanofibers must be expected for NADES/Sp- and PVA-based emulsions, and for the W/Sp and PVA emulsions. 

It is important to note that, for the NADES 40/60 and NADES 50/50 nanofibers, since no nanofibers were formed, the diameter of nanofibers was set at zero for the PCA and Pearson correlation test. 

Therefore, for future optimization of NADES/Sp electrospinning, the viscosity seems to be more relevant than the conductivity of the emulsions in the formation of thick nanofibers.

**Figure 11:**
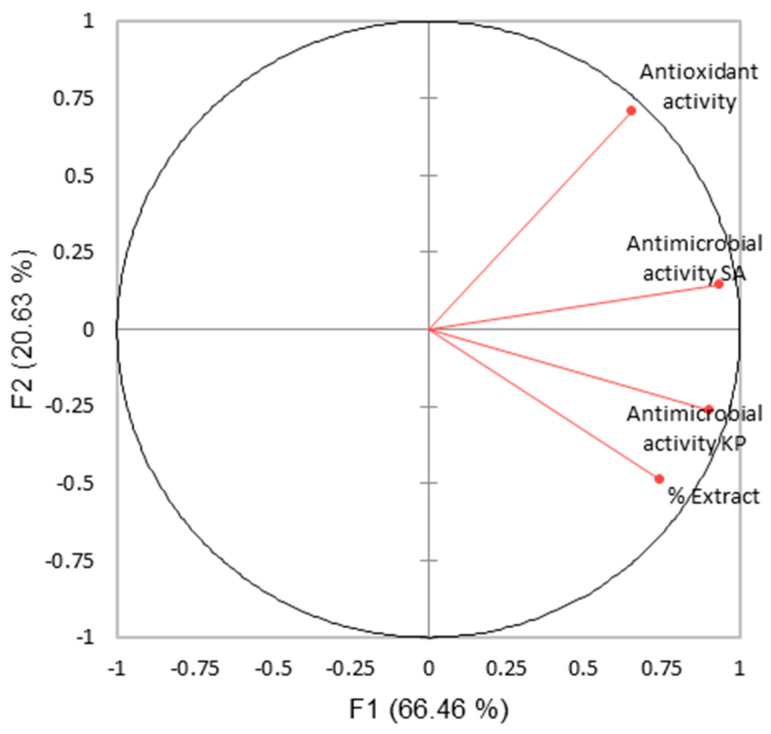
PCA biplot for the extract content, and the antioxidant and antimicrobial activities.

A second PCA was conducted using the extract content as the emulsion independent variable and the results obtained for the antioxidant and antimicrobial activities as the correlated dependent variables. The Pearson correlation matrix is shown in Table 6, and the PCA biplot is shown in Figure 11.

Pearson correlation test did not show significant correlations between the content of extract in the electrospinning emulsion and the antioxidant, and antimicrobial activities of the nanofibers (for *p*-value = 0.05). Nonetheless the content of the extract was positively related to the antimicrobial activity against the *S. aureus* and *K. pneumoniae* bacteria. In addition, the antioxidant activity was positively correlated with the extract content in the emulsion, which was also expected, due to the antioxidant properties of the *S. platensis* extracts [45]. Despite the fact that the correlation between the extract content and the antioxidant activity of the nanofibers did not prove to be statistically significant, it could serve as a guide for future research, and for the optimization of the extraction process prior to the formation of the emulsions containing *S. platensis* extracts.

## 4. Conclusions and Future Perspectives

In this work, the electrospinning of emulsions containing *Spirulina platensis* extracts—obtained through a green extraction technique using a glucose/glycerol based NADES, water, and PVA as a co-spinning agent, was reported for the first time. The results obtained were promising, since smooth nanofibers were obtained for the W 5/95, W 20/80, NADES 5/95, and NADES 20/80 emulsions. Moreover, the NADES/Sp- and PVA-based nanofibers showed higher antioxidant and antimicrobial activities than those of the W/Sp- and PVA-based nanofibers. Thus, the NADES 20/80 nanofibers might be highly valuable for several applications (i.e., food packaging and textiles). 

Although the results were promising, there should be further investigation in order to optimize the formation of the nanofibers for higher extract content, as well as their antimicrobial and antioxidant properties. Nonetheless, this study also opens a novel and greener strategy for both the extraction of bioactive compounds and for solvent-free electrospinning, making electrospinning technology an extraordinary solution to provide sustainable materials aligned with UN sustainability goals.

For future work, the authors propose the determination of the surface tension of the electrospinning emulsions and the evaluation of the mechanical properties, thermal stability and biodegradability of the nanofibers in order to determine its use in several applications such as packaging, textile fabrics, and the pharmaceutical sector.

More importantly, all variables of the electrospinning technique (i.e., viscosity, polymer concentration, electrical conductivity, surface tension, applied voltage, distance from electrode to collector, volume feed rate, temperature, and humidity) must be optimized in order to reach better fiber formation and morphology. Furthermore, the main independent variables (i.e., temperature and time) of the extraction method should also be optimized in order to obtain higher yields of valuable pigments (i.e., chlorophylls, carotenoids, and phycocyanin) with antioxidant and antimicrobial properties. The authors propose the use of the Response Surface Methodology, or RSM, for both the electrospinning and the extraction optimization. Finally, biobased-polymers different from PVA could also be investigated.

## Figures and Tables

**Table 1:** Nomenclature used in the article.

Symbol	Description
W/Sp	Extract from water and UAE of *Spirulina platensis*
NADES/Sp	Extract from NADES and UAE of *Spirulina platensis*
PVA 9%	Electrospun nanofibers from a PVA 9% (*w*/*v*) emulsion
NADES 50/50	Electrospun nanofibers from an emulsion of 50% NADES/Sp extract and 50% PVA (*v*/*v*)
NADES 40/60	Electrospun nanofibers from an emulsion of 40% NADES/Sp extract and 60% PVA (*v*/*v*)
NADES 20/80	Electrospun nanofibers from an emulsion of 20% NADES/Sp extract and 80% PVA (*v*/*v*)
NADES 5/95	Electrospun nanofibers from an emulsion of 5% NADES/Sp extract and 95% PVA (*v*/*v*)
W 50/50	Electrospun nanofibers from an emulsion of 50% W/Sp extract and 50% PVA (*v*/*v*)
W 40/60	Electrospun nanofibers from an emulsion of 40% W/Sp extract and 50% PVA (*v*/*v*)
W 20/80	Electrospun nanofibers from an emulsion of 20% W/Sp extract and 80% PVA (*v*/*v*)
W 5/95	Electrospun nanofibers from an emulsion of 5% W/Sp extract and 95% PVA (*v*/*v*)

**Table 2:** Minimum inhibitory concentration (mg/mL) of *Spirulina platensis* extracts.

	MIC (mg/mL)	
Extract	*K. pneumoniae* (ATCC 4352)	*S. aureus* (ATCC 6538)
W/Sp	10.00	10.00
NADES/Sp	4.17	8.34

**Table 3:** Matches found by LabSolutionsIR software for all nanofibers produced.

Sample	Degree of Confidence	Corresponding Polymer
PVA 9%	835—Medium	PVA
W 50/50	773—Medium	
W 40/60	715—Medium	
W 20/80	801—Medium	
NADE 20/80	807—Medium	
NADE 40/60	826—Medium	Glycerol
NADE 50/50	834—Medium	

**Table 4:** Color analysis of nanofibers.

Sample	Apparent Color	L*	a*	b*	C*	h	Color Strength (K/S)
Collector	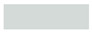	87.61	−0.44	1.22	1.30	109.94	34.76
PVA 9%	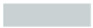	84.70	−0.74	−0.34	0.82	204.60	31.88
NADES 50/50	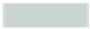	85.60	−1.64	0.62	1.76	159.44	32.96
NADES 40/60	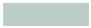	82.80	−4.29	1.95	4.71	155.49	30.49
NADES 20/80	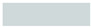	87.40	−1.35	0.34	1.39	165.88	34.61
W 50/50	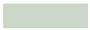	85.90	−3.30	6.10	6.93	118.44	33.48
W 40/60	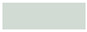	87.40	−1.53	2.71	3.11	119.36	34.70
W 20/80	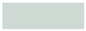	87.00	−2.24	2.43	3.31	132.64	34.32

**Table 5:** Pearson correlation matrix for emulsion properties: viscosity, conductivity, and diameter of the nanofibers produced with the emulsions prepared.

Variables	Conductivity	Viscosity	Diameter of Nanofibers
Conductivity	**1**	0.507	0.446
Viscosity	0.507	**1**	0.560
Diameter of nanofibers	0.446	0.560	**1**

Values in bold indicate significant correlations at significance level *p*-value = 0.05.

**Table 6:** Pearson correlation matrix.

Variables	% Extract	Antioxidant Activity	Antimicrobial Activity SA	Antimicrobial Activity KP
% Extract	**1**	0.260	0.508	0.654
Antioxidant activity	0.260	**1**	0.629	0.340
Antimicrobial activity SA	0.508	0.629	**1**	**0.841**
Antimicrobial activity KP	0.654	0.340	**0.841**	**1**

Values in bold indicate significant correlations at significance level *p*-value = 0.05.

## Data Availability

The data are fully displayed in the main text of this paper.

## Supplementary Materials

The following supporting information can be downloaded at: https://www.mdpi.com/article/10.3390/polym15061574/s1, Figure S1: UV spectrum for the extracts obtained in the NADES/Sp UAE extraction; Figure S2: UV spectrum for the extracts obtained in the W/Sp UAE extraction.
