# Supplementary material for: Natural Deep Eutectic Solvent Extraction of Bioactive Pigments from Spirulina platensis and Electrospinning Ability Assessment
Source: Polymers (Basel). 2023 Mar 22;15(6):1574. doi: 10.3390/polym15061574 (PMC10054781; doi:10.3390/polym15061574)
Supplement: Supplementary file 1 [file polymers-15-01574-s001.zip › polymers-2277466-supplementary.pdf]

Supplementary material

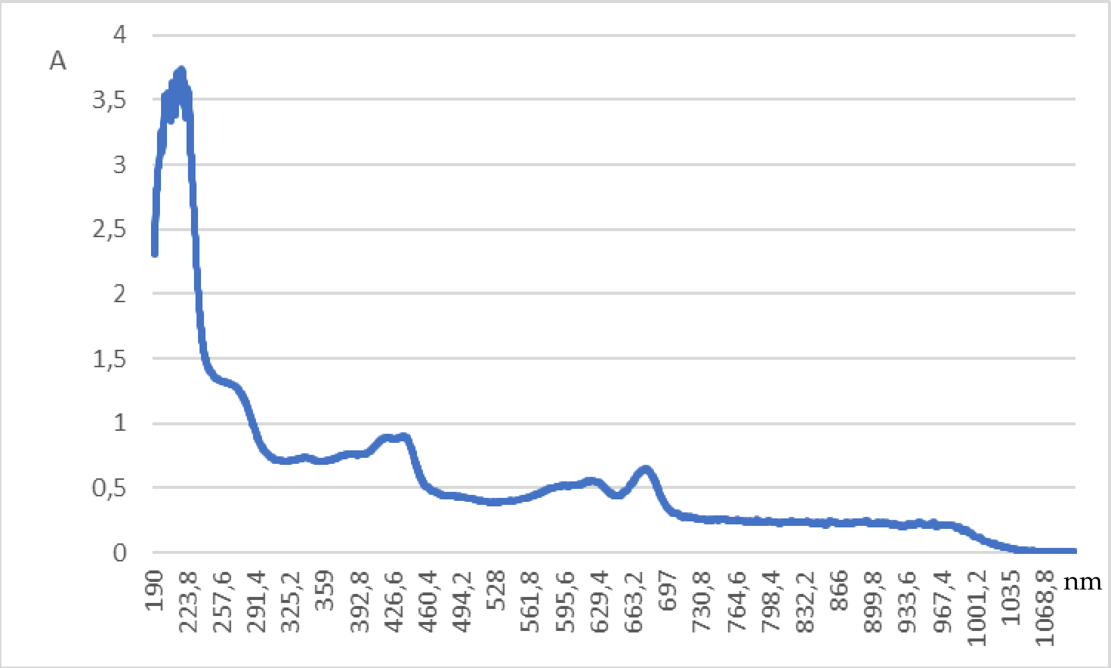

***Figure S1** – UV spectrum for the extracts obtained in the NADES/Sp UAE extraction.*

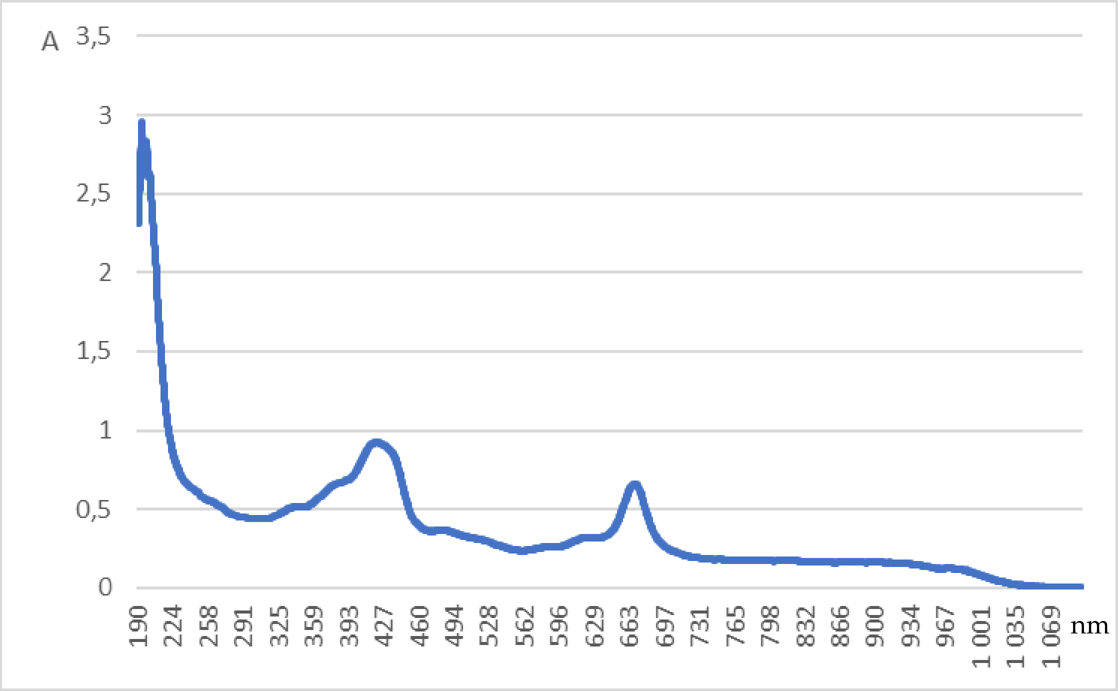

***Figure S2** – UV spectrum for the extracts obtained in the W/Sp UAE extraction.*
